# Oxidative Polymerization of Aniline on the Surface of Sisal Fibers (SFs) as Defluoridation Media for Groundwater

**DOI:** 10.1155/2024/6941567

**Published:** 2024-02-01

**Authors:** Tesfamariam Teklu

**Affiliations:** Department of Chemistry, Mekelle University, Mekelle, Tigray, Ethiopia

## Abstract

Chemical modification of sisal fibers via *in situ* oxidative polymerization of aniline was conducted to examine their defluoridation capacity for fluoride from drinking water. The effects of polyaniline modifications have shown significant changes on the chemical moieties and defluoridation capacity of sisal fibers (SFs). FTIR peaks at 1440 cm^−1^ and 1560 cm^−1^ revealed the presence of benzoid and quinoid structures together with sisal fiber (SF). Thermal profiles confirmed the enhancement of thermal stability of polyaniline-modified sisal fibers (PAniMSFs). SEM microstructure also proved the surface roughening of SFs as a result of polyaniline modifications. Optimal batch adsorption parameters (pH, contact time, adsorbent dose, and initial concentration) were found to be 5, 60 min, 1 g, and 10 mg/L, respectively. Adsorption kinetics proved that the removal of fluoride follows pseudo-second-order model (*K*_2_ = 0.18 g. (mg·min)^−1^), while the adsorption isotherm well described by the Langmuir and Freundlich model with an experimental adsorption capacity of 2.49 mg/g. Hence, modifications and improvements are required to reduce the amount of fluoride to a permissible level and enhance the longevity and activity of adsorbent materials.

## 1. Introduction

Fluoride is considered an essential micronutrient for the human body to prevent dental caries and mineralization of hard tissues [1]. Beyond the optimal level recommended by WHO, 1.5 mg/L, fluoride becomes toxic causing bone disease and mottling of teeth [2]. Many scholars have reported that continuous fluoride uptake damages the liver, kidney, and nervous system [3]. More than 200 million worldwide, of which about 8 million people living in the Ethiopian Rift Valley rely on groundwater and boreholes contaminated with fluoride as high as 33 mg/L [4, 5].

Volcanic rocks in the East African Rift Valley possess a large deposit of fluorapatite mineral (Ca_5_(PO_4_)_3_F) compared to analogous rocks in different areas. The Main Ethiopian Rift Valley is geologically unstable associated with rifting by hydrothermal energy and hot climate, which facilitates the solubility of natural rocks containing fluorapatite and other minerals [6]. Consequently, defluoridation at a point of use is a must to provide the public with safe drinking water.

Adsorption, a physicochemical method, is preferentially selected over precipitation, osmosis, and coagulation to remove excess fluoride from drinking water because it is simple, low cost, and easily regenerable [7]. Several adsorbent materials have been tested for similar purposes. Activated carbon possesses a high surface area which is considered as the most efficient adsorbent. However, production cost and regenerability restricted its comprehensive use [8]. Although the Nalgonda project was applied in different parts of the world, it was not socially accepted and finally abandoned [9]. Different minerals were also tested to remove fluoride from drinking water. However, the generation of sludge restricted their popularity [10].

Nowadays, composites and resins attract the attention of scientists to develop defluoridation media for drinking water. Polyaniline is rich in amine and imine polyfunctionalities [11], which makes it ideal for ion exchange and ion pair interactions. It also uses the cheapest monomer and the easiest to synthesize [12]. Different reports proved that polyaniline composites have shown appreciable defluoridation efficiencies from drinking water [13]. However, the limited processability in aqueous environments and formation of aggregation hampers the versatility of polyaniline which in turn reduces the adsorption efficiency.

Fibrous materials have emerged as substrates to alleviate the processability issues and formation of aggregation of sole polyaniline [14]. Sisal (*agave sisalana*) fiber is technically chosen as a substrate to carry out the active functionality of polyaniline, which is functionally rich in hydroxyl and carbonyls ready to make physicochemical attachments, mechanically fairly strong, and withstands the deterioration from saline water [15]. Furthermore, chemical polymerization of aniline on fibrous materials enhanced the distribution of amine and imine multifunctionalities responsible to capture fluoride ions [16].

Therefore, this work presented the surface modification of sisal fibers via *in situ* oxidative polymerization of aniline to evaluate their defluoridation capacity from drinking water using a fluoride analyser electrode.

## 2. Materials and Methods

### 2.1. Chemicals and Materials

The following chemical reagents were of analytical grade: aniline (99%), ferric chloride hexahydrate (98%), sodium fluoride (98%), hydrochloric acid (37%), sodium hydroxide pellet (extra pure), and sodium nitrate purchased from Merck Chemical Co. (Darmstadt, Germany) through a local company.

### 2.2. Experimental Procedures

Matured sisal leaves were harvested from Shafat, 10 km to the south of Mekelle, Tigray, northern Ethiopia, and washed using running water. Sisal fibers were extracted using soil retting by burying matured sisal leaves approximately 40 cm deep on the ground for three weeks in a moisturized soil [17]. The extracted fibers were carefully washed with distilled water and then air-dried in a shade for about two weeks. These fibers were shredded into pieces using a paper shredder. Then, these fibers were sieved based on their particle size by using the desired mesh numbers.

### 2.3. Polymerization of Aniline on Sisal Fibers (SFs)

Approximately, 10 g sisal fiber (SF) was soaked in 1 M acidified aniline. An equivalent amount of 1 M FeCl_3_.6H_2_O solution was poured into the mixture dropwise under continuous stirring [18]. The polymerization process was adjusted for 24 h at room temperature. After termination of the reaction, the polyaniline-modified fibers were filtered and washed thoroughly with excess dilute HCl solution and distilled water, respectively. Finally, it was oven-dried at a lower temperature.

### 2.4. Characterization of Polyaniline-Modified Sisal Fibers (PAniMSFs)

Screening of the chemical functionality of free sisal fibers (SFs) and polyaniline-modified sisal fibers (PAniMSFs) was performed using Fourier-transform infrared (FTIR) spectroscopy (Bruker Vector 22). They were put to a thermal experiment on a simultaneous thermal analyser (DSC-TGA, SDT-Q600) from 25°C to 600°C to examine the weight loss as a function of temperature. External surfaces of SFs and PAniMSF were monitored using scanning electron microscopy (SEM) (JSM-IT300LV, JEOL, USA) at 500x magnification equipped with energy-dispersive X-ray (EDS) spectroscopy.

### 2.5. Preparation of Fluoride (F^−^) Ion Solutions

A stock solution of 1000 mg/L fluoride ion was prepared by solubilizing 0.221 g NaF in 1 L distilled water. An intermediate solution of 100 mg/L was prepared by the dilution method, and other working solutions were prepared by further dilution to the desired concentrations (1, 5, 10, 15, and 20 mg/L) [19]. Blank and control solutions were prepared and performed throughout the experimental process.

### 2.6. Optimization of Batch Adsorption Parameters

#### 2.6.1. Effect of Solution pH

Approximately, 0.5 g PAniMSF was mixed with 200 mL of 10 mg/L fluoride solution by tuning the pH range from 1 to 10 by using 0.1 M HCl and 0.1 M NaOH solution for 50 min. The pH at the point of zero charge (pH_PZC_) was determined by using the pH drift method with minor modifications, by placing 0.1 g adsorbent in 100 mL of 0.01 M NaNO_3_ solution [20].

#### 2.6.2. Effect of Contact Time

About 0.5 g PAniMSF was mixed with 200 mL of 10 mg/L fluoride solution and stirred at the following time intervals (5, 15, 30, 45, 60, 75, 90, 105, 120, 150, and 180 min) at pH 5 and room temperature.

#### 2.6.3. Effect of Adsorbent Dosage

The following amount (0.25, 0.5, 1, 1.5, 2, 2.5, 3, 4, 5, and 6 g) of PAniMSF was added to 200 mL of 10 mg/L fluoride solution at pH 5, for 60 min, and stirred at room temperature.

#### 2.6.4. Effect of Initial Fluoride Concentration

An optimum amount of PAniMSF (adsorbent dosage of 1 g) was added into 200 mL of (1, 3, 5, 8, 10, 12, and 15 mg/L fluoride solution, respectively) and stirred at pH 5 for a duration of 60 min at room temperature. After equilibration and achievement of optimum points for all parameters, the solution was filtered through the Whatman filter paper. The residual fluoride solution was analysed by using a fluoride ion selective analyser (Hanna, HI552202, Romania).

Finally, the regeneration of adsorption materials was checked to test longevity and recyclability using dilute HCl solutions. All adsorption tests were conducted two times in triplicate measurements, and the reported amounts of fluoride ions are the mean of the experimental findings.

### 2.7. Column Adsorption

Adsorption capacity was also checked in the column experiment. A transparent glass column (internal diameter: 50 mm and length: 100 cm) was used for the continuous removal of fluoride from fluoride simulated solution. The bottom end of the column was sealed off with a sieve pore size less than 50 mm. The column was filled with 100 mm size of the adsorbent material. The known concentration of fluoride solution (10 mg/L) was continuously fed in the downward through the column. To investigate the effect of the flow rate, a series of experiments were conducted at four different flow rates (5, 10, 15, and 20 mL/min). The experiment was equipped with a stopper valve to control the flow rate. Then, the filtrate was collected for a fluoride analysis using the fluoride ion selective electrode.

## 3. Results and Discussion

### 3.1. Fourier-Transform Infrared (FTIR) Spectroscopy

**Figure 1:**
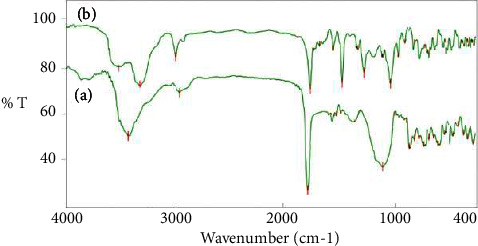
FTIR spectra of (a) sisal fibers (SFs) and (b) polyaniline-modified sisal fibers (PAniMSFs).

FTIR spectra of SF and PAniMSF are shown in Figure 1. The FTIR spectra of SF and PAniMSF possess common absorption bands beyond 3300 cm^−1^ that belong to the O-H [21] and N-H [22] stretching vibrations derived from cellulose and aniline, respectively. To some extent, peak broadening was observed when the polymerization of aniline occurred together with sisal fibers (Figure 1(b)). A very weak band at 2900 cm^−1^ belongs to C-H stretching vibrations of alkyl hydrocarbons derived from the fiber [23]. The FTIR peak which belongs to hydroxyl functionality has shifted to the lower band after adsorption of fluoride ion (Figure not included here). This can be justified by the facilitation of fluoride adsorption via substitution of hydroxyl groups [24].

The peak at 1730 cm^−1^ is peculiar to the stretching vibration of carbonyl (C=O) functionality of hemicellulose and lignin of sisal fibers (Figure 1(a)) [25]. However, there is a marked reduction of peak intensity during polymerization due to the interaction of N-H moieties with the existing system. Growth of polyaniline on SFs confirmed the appearance of two specific peaks at 1440 and 1560 cm^−1^ which represent polyamino functionalities, benzoid (-NH-B-NH-)_n_ and quinoid (-N = Q-N = )_n_ rings, respectively. These peaks signify the inclusion of polyaniline in its partially oxidized form [18, 26], which are totally absent in sisal fibers.

### 3.2. Thermogravimetry Analysis (TGA)

**Figure 2:**
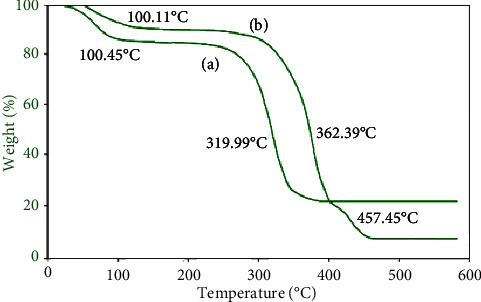
TGA thermogram curves of (a) sisal fibers (SFs) and (b) polyaniline-modified sisal fibers (PAniMSFs).

Thermal analysis is an essential method used to investigate the weight loss of a material with temperature. Thermal analysis (TA) of SF and PAniMSF is shown in Figures 2 A and B, respectively. The first degradation peak, which extends up to 100°C, belongs to mass loss due to evaporation of chemically and physically adsorbed moisture [27]. However, the mass loss is less for PAniMSF. A very sharp degradation profile which stretches up to 360°C represents the decomposition of cellulose, hemicellulose, and pectin derived from sisal fibers [28]. The last stage beyond 450°C inferred the degradation of polyaniline, which is absent in the sisal fiber (line A). Introduction of polyaniline further proved the enhancement of thermal stability of sisal fiber [25, 29].

### 3.3. Surface Morphology (SEM) Coupled with Energy-Dispersive X-Ray Spectroscopy (EDS)

**Figure 3:**
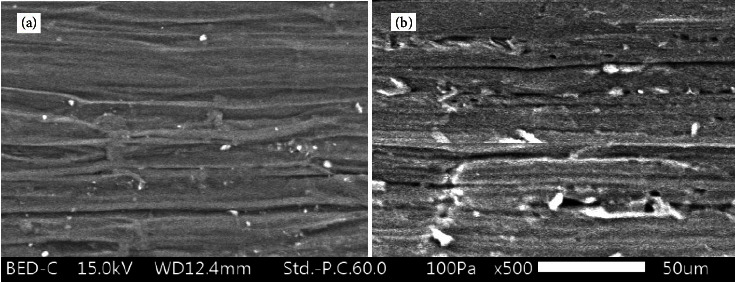
SEM microstructure of (a) sisal fibers (SFs) and (b) polyaniline modified sisal fibers (PAniMSFs).

**Figure 4:**
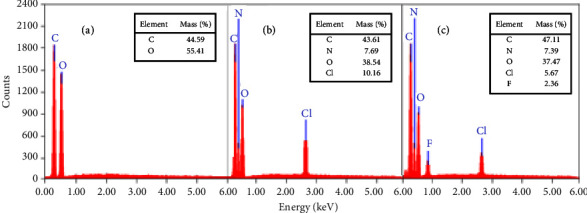
EDX measurements of (a) sisal fibers (SFs), (b) polyaniline-modified sisal fibers (PAniMSFs), and (c) polyaniline-modified sisal fibers (PAniMSFs) after adsorption of fluoride.

Morphological investigations of SF and PAniMSF were carried out by a scanning electron microscope (SEM) as presented in Figure 3. SEM micrographs proved the polymerization of aniline causes roughening the surface of the fiber (Figure 3(b)). The EDS spectra (Figure 4(a)) revealed strong peaks at CK*α*0.21 keV and Ok*α*0.51 keV which represent carbon and oxygen derived from sisal fibers. Additional peaks at NK*α*0.39 keV and ClK*α*2.62 keV (Figure 4(b)) belong to nitrogen and chlorine, suggesting the inclusion of the oxidized form of polyaniline during oxidative polymerization [30].

Furthermore, a new signal also appeared at Fk*α*0.94 keV (Figure 4(c)) showing the existence of considerable content of fluoride ions adsorbed on the PAniMSF surface [31] after the fluoride adsorption experiment. This confirmed that the PAniMSF surface was successful for fluoride capture. Mass of elemental oxygen showed a reduction trend after polymerization and adsorption of fluoride occurred. Probably, this could be evidence that oxygen-bearing species are replaced by fluoride ions during the adsorption process [24].

### 3.4. Batch Adsorption Study of Fluoride

#### 3.4.1. Effect of Solution pH

**Figure 5:**
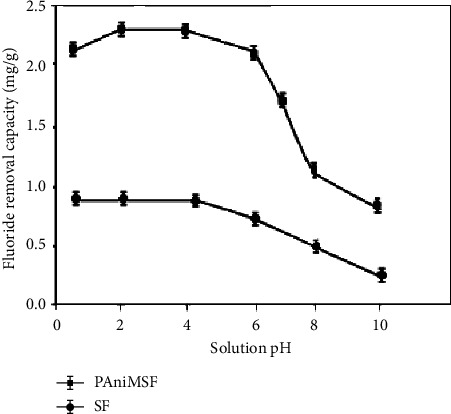
Effect of solution pH on defluoridation of water (*C*_*o*_: 10 mg/L, adsorbent dose: 0.5 g, and contact time: 50 min) at room temperature.

A series of experiments were performed at the pH range from 1 to 10 to evaluate the defluoridation capacity of PAniMSF, and the findings are presented in Figure 5. Indeed, the surface chemistry of adsorption materials is highly sensitive to little change in pH. The experimental study confirmed defluoridation was favoured at the acidic pH range. Protonation of nitrogen due to polymerization of aniline enhanced the electrostatic interactions between amine and imine surfaces with fluoride ions in aqueous solution. Furthermore, fluoride ions can easily displace a highly polarizable chloride ion which exists on the surface and interface of polyaniline as a counter anion [32].

The fluoride uptake capacity increased steadily (up to 2.21 mg/g) when the solution pH began to rise up from 1 to 4. In acidic media, both hydronium and chloride ions influence the surface chemistry of adsorption boosting protonation and doping, respectively. Moreover, such species can support the formation of cationic radicals favouring fluoride exchange [33]. Maximum defluoridation of fluoride was achieved at pH 5 (pH < pHpzc = 6.08) where the surface charge of the adsorbents remained positive [34]. At extreme acidity, the amount of fluoride uptake was reduced due to the formation of weak fluoro acids and complex species (HF, HF_2_^−^, and F^−^) which can affect the accessibility and mode of interaction of fluoride [32].

A further increase in solution pH (6 to 10) resulted in a sharp reduction of fluoride uptake (1.93 mg/g to 0.67 mg/g) due to the competition for active sites between hydroxyl groups and fluoride ions [33]. Furthermore, a reduction in adsorption capacity beyond pHpzc (pHpzc = 6.08) is rationalized by electrostatic repulsion between the fluoride ion and the surface of the adsorbents which is dominantly charged negatively [35].

It is important to note that SFs removed a substantial amount of fluoride although the removal capacity was not comparable to polyaniline-modified ones. The hydroxyl functionalities of sisal fibers can interact with hydronium ions in the solution to form positively charged surfaces leading to fluoride uptake. Moreover, other weak van der Waals interactions may occur between fluoride and the sisal surface [36]. Different scholars have applied cellulose, chitin, and chitosan as adsorption media due to the availability of hydroxyl functionality through H-bonding and other van der Waals interactions. However, their adsorption capacities were minimal in which modification and activation to acquire amines, carbonyl, and hydroxyl in their anatomical chains enhanced removal efficiency [31, 37].

#### 3.4.2. Effect of Contact Time and Adsorption Kinetics

**Figure 6:**
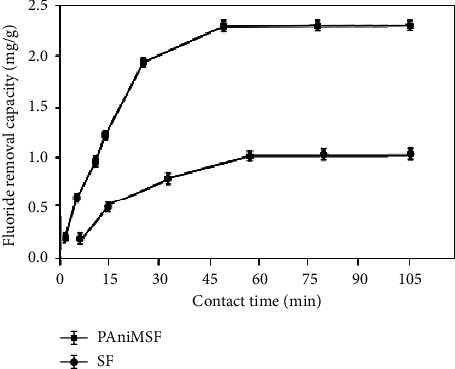
Effect of contact time on defluoridation of water (*C*_*o*_: 10 mg/L, pH: 5, and adsorbent dose: 0.5 g) at room temperature.

The effect of contact time on the removal of fluoride ion using PAniMSF is presented in Figure 6. Findings of adsorption capacity confirmed that the removal of fluoride was fast enough up to the first 45 min. Approximately, 2.3 mg/g of the fluoride ion was defluoridated in a short time. This may be justified by the availability of excess active sites and the high concentration gradient at the initial stage. It can be visualized (Figure 6) that an equilibrium was achieved beyond 60 min. Once equilibrium was achieved, the rate of defluoridation remained almost the same throughout the process due to further reduction of the number of vacant sites and the amount of fluoride ions in the solution [38]. Sisal fibers also followed similar adsorption trends. However, the adsorption capacity is much lower than polyaniline modification.

In order to validate the experimental data and forecast the rate of fluoride uptake on the surface and interface of PAniMSF, Lagergren pseudo-first-order [39] and pseudo-second-order [40] models are applied. The linear versions of the Lagergren pseudo-first- and second-order expressions are shown in equations (1) and (2), respectively:

(1) $$\begin{array}{c} \ln\;\left(q_{e}-q_{t}\right)=\ln\;{\;q}_{e}-K_{1}t, \end{array}$$

(2) $$\begin{array}{c} \frac{t}{q_{t}}=\frac{1}{K_{2}{qe}^{2}}+\left(\frac{1}{q_{e}}\right)t, \end{array}$$

(3) $$\begin{array}{c} q_{e}=\left(\frac{C_{o}-C_{e}}{m\;}\right)V, \end{array}$$

where *q*_*e*_ and *q*_*t*_ (mg/g) are the adsorption capacities at equilibrium and time (*t*, min), respectively, *K*_1_ (1/min) and *K*_2_ (g/(mg·min)) are the rate constants for the Lagergren pseudo-first-order and pseudo-second-order models, respectively, *C*_*o*_ and *C*_*e*_ are the initial and residual concentrations of fluoride (mg/L) at equilibrium, respectively, *m* is the mass of adsorbent (*g*), and *V* is the total volume of a solution (*L*).

**Figure 7:**
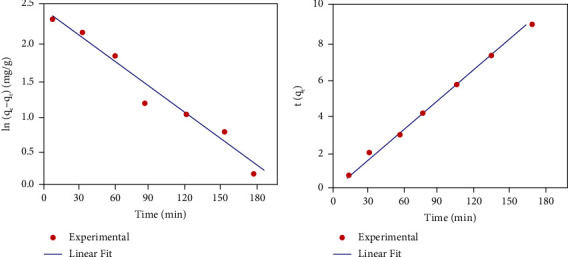
Kinetic models of (a) Lagergren pseudo-first-order and (b) pseudo-second-order adsorption of fluoride (*C*_*o*_: 10 mg/L, pH: 5, and adsorbent dose: 1 g) at room temperature.

Rate constant (*K*_1_) and adsorption capacity (*q*_*e*_) at equilibrium are computed the slope and intercept of the plot of ln (*q*_*e*_ − *q*_*t*_) against time (*t*) from the linearized equation of the Lagergren pseudo-first-order model (Figure 7(a)). The graph is largely deviated from a straight line (regression coefficient, *R*^2^ = 0.91). The calculated adsorption capacity (*q*_*e*_, cal = 9.14 mg/g) determined from the graph (Table 1) differs significantly from the experimental adsorption capacity (*q*_*e*_, exp = 2.49 mg/g) (calculated using equation (3)). These results demonstrated that the Lagergren pseudo-first-order kinetics is not appropriate for fluoride adsorption on PAniMSF.

Rate constant (*K*_2_) and adsorption capacity (*q*_*e*_) are calculated from the intercept and slope of the plot of *t*/*q*_*t*_ against time *t* from the linearized equation of pseudo-second-order kinetics (Figure 7(b)). The graph is satisfactorily fitted to a straight line (regression coefficient, *R*^2^ = 0.98). The calculated adsorption capacity (*q*_*e*_, cal = 2.65 mg/g) is approximately equal to the experimentally determined adsorption capacity (*q*_*e*_, exp = 2.49 mg/g). These results suggest that pseudo-second-order mechanism is favourable for the adsorption of fluoride. Defluoridation onto PAniMSF occurring for a short period may prove the existence of strong ionic interactions between the positively charged polyaniline surface and fluoride ions, referred as chemical adsorption [41]. Rate constants (*K*_1_ and *K*_2_) and adsorption capacity (*q*_*e*_) calculated from the plots of Lagergren pseudo-first- and second-order kinetics are shown in Table 1.

#### 3.4.3. Effect of Adsorbent Dosage

**Figure 8:**
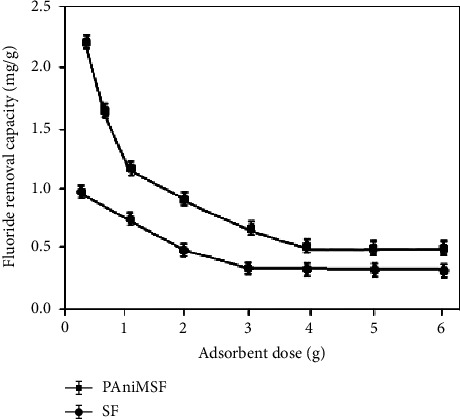
Effect of adsorbent dose on defluoridation of water (*C*_*o*_: 10 mg/L, pH: 5, and contact time: 60 min) at room temperature.

To achieve greater adsorption capacity, an adsorbent dose of 0.25 g to 6 g was used to defluoridate 10 mg/L of fluoride solution (Figure 8). Fluoride uptake capacity was enhanced with a lessening in adsorbent dosage due to the accessibility of a higher number of fluoride species per unit mass of adsorbents [42]. The ability to adsorb fluoride was decreased from 2.32 mg/g (solid/liquid ratio = 1.25 g/L) to 1.23 mg/g (solid/liquid ratio = 5 g/L). Figure 8 illustrates that a further increase in the adsorbent dose (beyond 3 g) has little effect on the defluoridation capacity due to the very low equilibrium concentration of fluoride [43]. Sisal fibers, on the other hand, only managed to reach a maximum of 0.97 mg/g during the trial, and when the dose of adsorbent was increased, they further decreased to 0.25 mg/g.

#### 3.4.4. Effect of Initial Concentration and Adsorption Isotherms

Results of adsorption capacity for initial fluoride concentrations are shown in Table 2. Fluoride take-up has ceaselessly increased with an increment in the sum of fluoride, whereas the amount of adsorbent remained unchanged and solidness accomplished. This can be legitimized by the presence of a higher number of fluoride than the number of adsorbents which consequently goes to saturation [44]. However, polyaniline modification boosted the adsorption capacity (*q*_*e*_) of sisal fibers (PAniMSF: 2.48 mg/g versus 0.82 mg/g of SF) which confirmed the inclusion of polyaniline moieties enhanced the surface chemistry of sisal fibers.

Langmuir and Freundlich isotherm models are frequently utilized to characterize the adsorption behavior of adsorbents in the liquid phase. The Langmuir isotherm is utilized to measure the arrangement of monolayer adsorption on homogeneous adsorbent sites [45] with the thought that all adsorption destinations are indistinguishable and there is no interaction among adsorbed species on adjoining binding sites. The Freundlich isotherm considers adsorption taken at heterogeneous adsorption sites leading to multilayer formation [46]. The linearized forms of Langmuir and Freundlich isotherm models appear in equations (4) and (5), respectively:

(4) $$\begin{array}{c} \frac{C_{e}}{q_{e}}=\frac{1}{q_{m}.K_{L}}+\frac{C_{e}}{q_{m}}, \end{array}$$

(5) $$\begin{array}{c} \log\;{\;q}_{e}=\log\;K_{F}+\left(\frac{1}{n_{F}}\right)\log\;{\;C}_{e}, \end{array}$$

where *q*_*m*_ (mg/g) and *K*_*L*_ (L/mg) are Langmuir constants which belong to the maximum monolayer adsorption capacity and energy of adsorption, respectively, and *K*_*F*_ (mg/g) and *n*_*F*_ represent the multilayer adsorption capacity and the degree of dependence of adsorption at equilibrium concentration, respectively.

**Figure 9:**
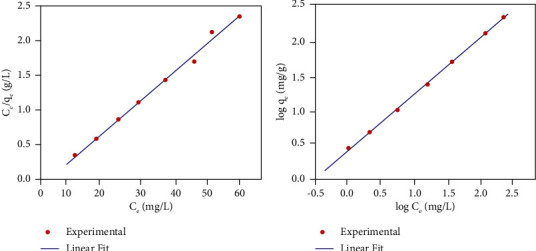
Equilibrium isotherms of (a) Langmuir and (b) Freundlich models of fluoride adsorption (pH: 5, adsorbent dose: 1 g, and contact time: 60 min) at room temperature.

The empirical values of Langmuir adsorption constants (*q*_*m*_ and *K*_*L*_) are computed from the slope and intercept of the linear plot of *C*_*e*_/*q*_*e*_ against *C*_*e*_, respectively. Essentially, Freundlich isotherm parameters (*K*_*F*_ and *n*_*F*_) are calculated from the intercept and slope of the plot of log *q*_*e*_ versus log *C*_*e*_ relationship. The isotherm data are assessed by utilizing Langmuir and Freundlich isotherm models, and the corresponding results are shown in Figures 9(a) and 9(b), respectively. The isotherm parameters calculated from the slope and intercept of the respective linear plots are given in Table 3.

The regression coefficients of both Langmuir and Freundlich isotherms have palatably fitted to the straight lines for adsorption of fluoride ions in spite of the fact that minor variations exist. The highest adsorption capacity (*q*_*m*_ = 3.1 mg/g) calculated from the Langmuir isotherm graph showed an approximate value with the experimental adsorption capacity (*q*_*e*_, exp = 2.49 mg/g). Values of the separation factor (*R*_*L*_) (computed utilizing equation (6)) are all found within the range 0 < *R*_*L*_ < 1 [47] which suggests the favourability of the equilibrium state. The adsorption intensity of the Freundlich isotherm lies within the favourable range 0 < 1/*nF* < 1 [48]. Hence, both isotherm models fitted well for the adsorption of fluoride which includes physicochemical intuitive on the adsorbent surfaces. SEM microstructures (Figure 3) further demonstrated the occurrence of a nonuniform adsorbent surface, particularly when the adsorption cycles repeated:

(6) $$\begin{array}{c} R_{L}=\left(\frac{1}{1+K_{L}.C_{o}\;}\right). \end{array}$$

### 3.5. Comparison of Adsorption Capacities of PAniMSF with Other Adsorbents

Comparison of adsorption capacity of PAniMSF with other adsorption materials is troublesome due to the variety of experimental conditions. However, their execution can be generally assessed using adsorption capacity. Thus, the values of adsorption capacity of PAniMSF have appeared as average performance compared to the literature values (Table 4). Moreover, defluoridation includes either an ion-exchange mechanism or an electrostatic mode of interaction or both at a time to expel fluoride from water [50]. However, data on the nature of adsorbents (such as particle size, porosity, and dose), solution pH, and dosage of pollutants are either missed or totally distinctive in context.

### 3.6. Regeneration Studies

Adsorption is financially feasible. Consequently, any adsorbent material ought to be effortlessly recovered and reused as numerous times as conceivable [35]. Recovery tests were carried out using diverse concentrations of HCl solution as a desorbing agent. The adsorption test was conducted on the recovered adsorbent materials up to the seventh cycle with a small reduction in the adsorption performance. However, adsorption performance of the recovered adsorbents began to decrease essentially after the sixth adsorption cycle.

### 3.7. Column Study

**Figure 10:**
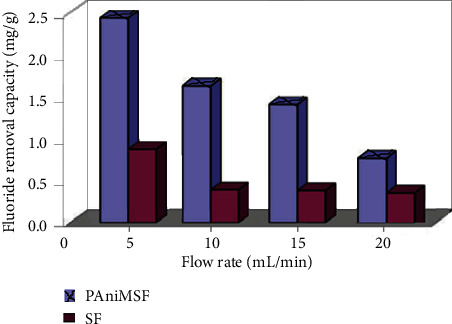
The effect of the flow rate on defluoridation of water (*C*_*o*_: 10 mg/L, pH: 5, and adsorbent dose: 1 g) at room temperature.

As adsorption is a time subordinate process, the impact of the flow rate on the adsorption of fluoride was explored by changing the flow rate from 5 to 20 mL/min, pH 5, and a dose of 1 g, and the corresponding results are shown in Figure 10. Adsorption capacity has shown a lessening pattern with an increasing flow rate due to the reduction in contact time between fluoride particles and adsorbents. However, lower flow rates give the chance to create superior adsorption contact, and thus, the defluoridation process is brought to an end. Defluoridation capacity of SFs (used as control) was substantially lower than PAniMSF. Adsorption capacity of sisal fibers (0.97 mg/g for 10 mg/L) is lower than the reported value of chitin (3.4 mg/g for 5 mg/L) [51] although the flow rate and initial fluoride concentration are different.

## 4. Conclusions

This study presents the development of defluoridation media from drinking water using sisal fibers modified by polyaniline via *in situ* oxidative polymerization of aniline. FTIR, TGA, and SEM-EDX studies revealed that sisal fibers are chemically modified using polyaniline. FTIR peaks at 1440 and 1560 cm^−1^ confirmed benzoid (-NH-B-NH)_n_ and quinoid (-N = Q-N = )_n_ amino functionalities of polyaniline exist together with the surface and interface of sisal fibers. Detection of particular peaks at NK*α*0.39 keV and ClK*α*2.62 keV from EDX spectra belongs to nitrogen and chlorine which proved the introduction of polyaniline in the partially oxidized and doped state.

Last, the fluoride uptake capacity was evaluated in batch and column experiments after optimization of adsorption parameters. The corresponding experimental results proved polyaniline modification showed appreciable adsorption capacity, 2.49 mg/g. Adsorption removal of fluoride ions followed pseudo-second-order kinetics, while both isotherms Langmuir and Freundlich fitted best to the experimental data.

To sum up, PAniMSFs have the potential to remove fluoride from drinking water when the adsorption parameters are fully optimized and monitored.

## Figures and Tables

**Table 1:** Lagergren pseudo-first- and pseudo-second-order kinetic parameters of fluoride ion adsorption at room temperature.

*C*_*o*_(mg/L)	*Lagergren pseudo-first-order*			*q*_*e*_, exp (mg/g)	Pseudo-second-order		
	*R* ^2^	*q*_*e*_, cal (mg/g)	*K*_1_(1/min)		*R* ^2^	*q*_*e*_, cal (mg/*g*)	*K*_2_(g/mg. min)
10	0.91	6.14	0.02	2.49	0.98	2.65	0.18

**Table 2:** The amount of fluoride ions adsorbed (*q*_*e*_, mg/g) by PAniMSF (pH: 5, adsorbent dose: 1 g, and contact time: 60 min) at room temperature.

Fluoride ion, *C*_*o*_(mg/L)	*Adsorption capacity (q*_*e*_ *, mg/g)*	
	PAniMSF	SF
1	0.89	0.26
3	1.08	0.42
5	1.26	0.68
8	2.44	0.81
10	2.48	0.82
12	2.49	0.86
15	2.48	0.87

^*∗*^The *q*_*e*_ values of PAniMSF and SF for fluoride concentration at 10 mg/L are 2.48 and 0.82 mg/g, respectively.

**Table 3:** Langmuir and Freundlich isotherm parameters of fluoride ion adsorption at room temperature.

*C*_*o*_(mg/L)	*Langmuir isotherm*				*Freundlich isotherm*		
	*R* ^2^	*K*_*L*_(L/mg)	*R*_*L*_	*q*_*m*_(mg/g)	*R* ^2^	*K*_*F*_(mg/g)	*n*_*F*_
10	0.97	0.51	0 < *R*_*L*_ < 1	3.1	0.99	0.83	0 < 1/*nF* < 1

**Table 4:** Summary of fluoride uptake capacity of polyaniline-based adsorbents.

Adsorbent	Adsorption capacity (mg/g)	Isotherm	Reference
Doped polyaniline	0.78	Freundlich	[13]
Polyaniline/polypyrrole	3.73	Freundlich	[49]
Polyaniline*/*montmorillonite	2.3	Freundlich	[30]
Polyaniline*/*alumina	6.6 mg/g	Freundlich/Langmuir	[47]
Polyaniline*/*chitosan	5.9	Freundlich/Langmuir	[50]
Polyaniline/sisal fiber	2.49	Freundlich/Langmuir	This study

## Data Availability

No additional data or supplemental materials are available for this paper.
